# Cellular and molecular changes in the skin driving increased nociception and pain during burn injury and repair

**DOI:** 10.3389/fpain.2026.1797161

**Published:** 2026-07-02

**Authors:** Chiara Nappi, Francisco J. Taberner

**Affiliations:** Instituto de Neurociencias, CSIC/UMH, Sant Joan, d’Alacant, Spain

**Keywords:** burn injury, immune-neuronal signaling, inflammation, inflammatory pain, neuropathic pain, nociception, pain, thermal injury

## Abstract

Burn injuries are among the most frequent traumas associated with human activity and primarily affect the skin. Unlike other forms of tissue injury, burns produce a unique combination of extensive tissue destruction, rapid release of damage-associated signals, and profound alterations in cutaneous architecture. These injury-specific features generate a robust inflammatory environment and early neuroimmune activation that can transition into neuropathic pain. Together, they drive marked peripheral sensitization and, in some cases, evolve into pain chronification, substantially affecting long-term quality of life in burn survivors. This review synthesizes current evidence on how thermal injury reshapes the function of key cutaneous cell populations, including keratinocytes, epidermal stem cells, melanocytes, fibroblasts, and immune cells, and how these changes modulate nociceptor activity through inflammatory, neurotrophic, and neuroimmune pathways. Furthermore, we describe how burn-induced cellular dysregulation contributes to peripheral sensitization across the continuum of wound healing, from acute inflammation to tissue repair. By integrating emerging mechanistic insights, this review highlights therapeutic targets aimed at minimizing subacute pain and preventing or mitigating chronic pain after burn injury.

## Graphical Abstract

**Figure:**
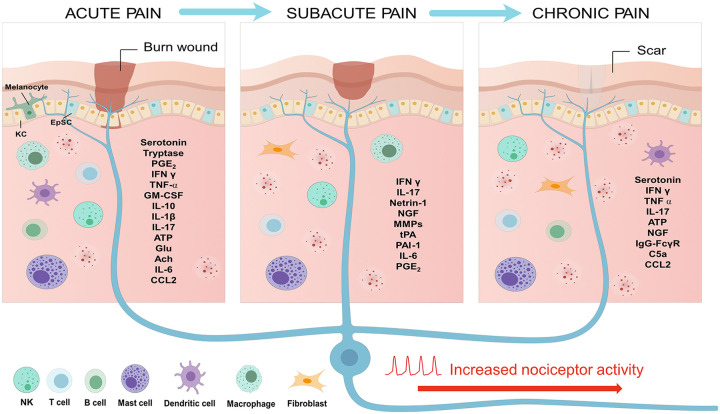
Following a burn injury, both epidermal and immune cells shape the evolving neuroimmune microenvironment, driving nociceptive sensitization and potentially leading to pain chronification. In the acute phase, keratinocytes and infiltrating immune cells, including mast cells, macrophages, dendritic cells, and NK cells, release pro-inflammatory mediators such as PGE2, TNF-α, and IL-1β, initiating nociceptor sensitization. During the subacute phase, fibroblasts, epidermal stem cells, and immune cells secrete neurotrophic and remodelling factors, including NGF, Netrin-1, and MMPs, which support tissue repair while may facilitate the transition toward chronic pain. In some individuals, even after scar formation, persistent activity of both immune and epidermal cells, along with sustained release of mediators such as NGF, IL-17 and C5a, maintains neuronal hyperexcitability and a state of chronic pain.

## 1 Introduction

Burns are among the most common injuries associated with human activity, affecting millions of people each year. The World Health Organization (WHO) estimates that approximately 180,000 deaths occur annually due to burn injuries, making them one of the leading causes of injury-related mortality (1). Non-fatal burns are a major cause of prolonged hospitalization, disfigurement, and disability, and they often present excruciating pain, resulting in psychosocial consequences.

Beyond the personal impact, burns impose a substantial economic burden. They remain among the most expensive traumatic injuries due to the complexity of medical care and the prolonged rehabilitation they require. In the United States, more than 410,000 burn injuries are reported each year, with approximately 40,000 requiring hospitalization and an estimated average cost of US $88,218 per patient (1). European data similarly highlight the magnitude of this issue: the annual incidence of severe burns ranges from 0.2 to 2.9 per 10,000 inhabitants, with hospitalization costs estimated at approximately €26,540 (2). When indirect costs, such as lost productivity, long-term disability, and the emotional impact on patients and families, are included, the economic burden of burn injuries increases.

The personal and economic burden of burn injuries is closely linked to their severity. Severity depends on multiple factors, including temperature and duration of exposure, burn depth, total body surface area involved, mechanism of injury (e.g., flame or scald), anatomical location, and patient-related characteristics such as age and comorbidities (3). Despite major advances in surgical techniques and intensive care that have significantly reduced mortality and improved outcomes, burn injuries still frequently lead to serious complications, including infection and sepsis, hypertrophic scarring, contractures, and long-term pain (3, 4). Pain during the healing process is extremely disabling and often persists long after wound closure and scar maturation. In a substantial proportion of patients, this chronic pain exhibits neuropathic features or evolves into neuropathic pain, characterized by symptoms such as burning, tingling, and electric shock-like sensations (5).

This review focuses on a critical aspect of thermal burn pathophysiology: the contribution of diverse cell populations in the skin to the modulation of nociception throughout the course of wound healing with particular emphasis on the acute burn response, where cellular mechanisms are best characterized. Beyond the well-established role of immune cells in initiating inflammation and shaping nociceptive responses, emerging evidence highlights the involvement of keratinocytes, epidermal stem cells, melanocytes, and fibroblasts in neuroimmune crosstalk and nociceptor sensitization. By integrating current knowledge on both immune and non-immune cellular contributors, this review aims to provide a comprehensive mechanistic framework for understanding how cellular dynamics influence nociception from the acute inflammatory phase through the reparative stages of healing. Such insights are essential for developing targeted therapies that not only accelerate wound closure but also alleviate chronic pain in burn survivors.

### 1.1 Inflammation in burn injuries

The inflammatory response is a defining feature of burn pathophysiology. Its spatial and temporal characteristics are strongly influenced by the unique zonal architecture of burn wounds, which comprises areas of coagulation, stasis, and hyperemia (3, 6, 7). This architecture not only determines the distribution of tissue damage but also guides immune cell recruitment and cytokine gradients, which are critical for initiating tissue repair and preparing the wound bed for regeneration (8). Compared with other skin injuries, burns elicit an intense and prolonged inflammatory phase, lasting from several days to weeks depending on injury depth and severity (3). Indeed, burn patients exhibit elevated levels of cytokines, chemokines, acute-phase proteins, and a hypermetabolic response, distinguishing burn-induced inflammation from that observed in surgical wounds or superficial abrasions (9).

In moderate to severe burns, inflammation can become dysregulated, leading to systemic complications including sepsis, multi-organ failure, and excessive fibrosis (3, 10). Dysregulated inflammatory cascades may also drive a catabolic state, delay recovery, and promote scarring (11, 12). Additionally, aberrant neuroimmune interactions can result in chronic pain syndromes that persist long after wound closure. Estimates indicate that 30%–50% of burn survivors develop chronic pain, often with a neuropathic component resulting from nerve injury, aberrant regeneration, and persistent inflammation (13, 14). The prolonged presence of inflammatory mediators and immune cells in the wound microenvironment is a key factor in the development of burn-associated neuropathic pain, which can be resistant to conventional analgesics.

### 1.2 Immune cell alterations and nociceptive dysregulation in burn injury

**Figure 1:**
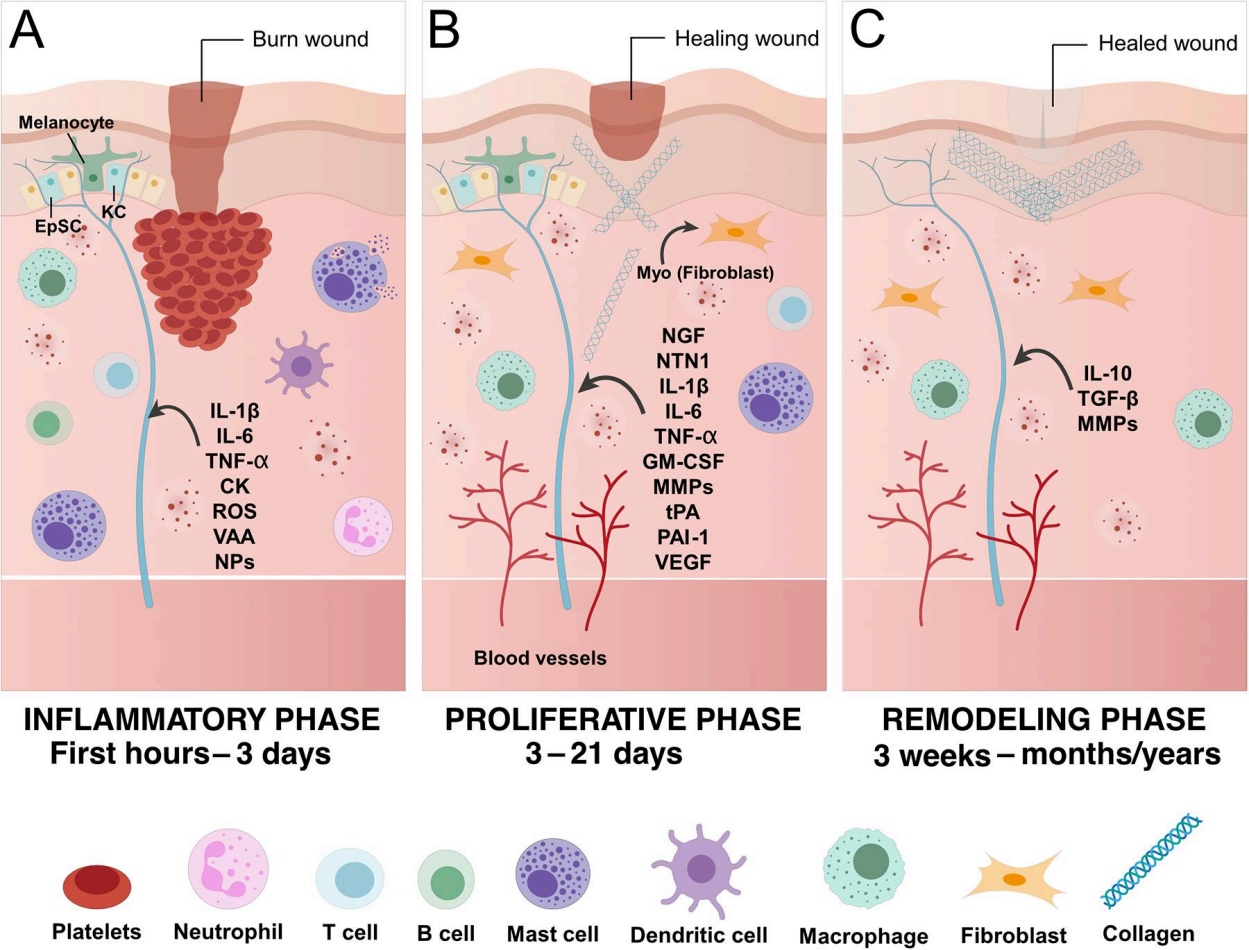
Immune cell contributions to nociceptive sensitization during burn wound healing. **(A)** Inflammatory phase (days 0–3): characterized by fibrin clot formation and the recruitment of neutrophils and macrophages for debris removal and secretion of pro-inflammatory cytokines (e.g., TNF-α, IL-1). **(B)** Proliferative phase: fibroblasts mediate the deposition of type III collagen and the formation of granulation tissue. Concurrently, re-epithelialization occurs, and vascular endothelial growth factor (VEGF) promotes neovascularization. **(C)** Remodeling phase: matrix maturation characterized by the replacement of type III collagen with organized type I collagen bundles, together with myofibroblast-induced wound contraction.

Burn injuries rapidly trigger a complex acute inflammatory response (Figure 1). Extensive tissue damage, and in some cases, microbial contamination, induces immediate activation of the innate immune system. In this context, damage-associated molecular patterns (DAMPs) released from necrotic cells, together with signals from activated tissue-resident immune cells, act as endogenous danger cues (15, 16). When infection is present, pathogen-associated molecular patterns (PAMPs) further amplify the inflammatory cascade (17–20). These molecular patterns are recognized by sentinel immune cells, including macrophages, mast cells, and dendritic cells, through pattern recognition receptors (PRRs) including Toll-like receptors (TLRs). Activation of TLRs initiates intracellular signaling pathways that drive the secretion of pro-inflammatory cytokines (e.g., TNF-α, IL-1β, IL-6, IL-8), chemokines, vasoactive amines, neuropeptides, reactive oxygen species (ROS), and lipid mediators derived from arachidonic acid (21, 22).

Additionally, burns rapidly disrupt vascular integrity in both macro- and microvessels of the affected tissue, producing plasma protein extravasation, interstitial edema, and fluid loss. This vascular dysfunction is a hallmark of burn physiology and contributes to burn shock and local ischemia (23). Thermal trauma also rapidly activates platelets, which release cytokines, chemokines, and microRNAs and form platelet–leukocyte aggregates that facilitate inflammatory cell recruitment (24, 25). In turn, the cytokines and chemokines released in the wound microenvironment activate endothelial cells, leading to the upregulation of adhesion molecules such as selectins, ICAM-1, and VCAM-1, which support neutrophil rolling, adhesion, and transmigration (26). Recent evidence also implicates pericytes as active regulators of leukocyte diapedesis, guiding neutrophils through permissive venular gaps and modulating interstitial migration programs (27). Together, these mechanisms promote the intense leukocyte infiltration that characterizes the acute inflammatory phase of burn injury.

Temporally, immediately after burn, mast cells degranulate, releasing mediators that initiate leukocyte recruitment. Thereafter, resident dendritic cells are activated within minutes to hours, sensing DAMPs through pattern-recognition receptors and amplifying the early inflammatory response by producing cytokines and chemokines that help guide incoming immune cells (7). Within hours, neutrophils become the dominant infiltrating population, followed sequentially by monocytes/macrophages, natural killer (NK) cells, and additional recruited dendritic cells. This coordinated early response shapes an inflammatory milieu that profoundly affects nociceptor excitability (Figure 1). Immune–neural crosstalk, driven by cytokines, neuropeptides, and modulation of ion channels such as TRPV1 and TRPA1, underlies peripheral sensitization and central amplification of burn-induced nociceptive signaling (28–30). The specific contributions of each immune cell type to this neuroimmune interaction are detailed in the sections below.

#### 1.2.1 Mast cells

Mast cells are tissue-resident immune sentinels distributed throughout the dermal layer, acting as first responders to the insult (31). Following thermal injury, these cells undergo rapid degranulation, releasing an array of bioactive mediators that initiate tissue repair while simultaneously contributing to burn-related acute and chronic pain (32).

Burns evoke a transient increase in immunoglobulin levels in the affected skin. Notably, IgE, the canonical activator of mast cells, is not reliably elevated in patients with low- to moderate-severity burns, indicating that IgE binding to the high-affinity Fc*ε*RI receptor is unlikely to be the primary pathway driving mast-cell activation in these cases. However, elevated IgE levels have been detected in serum and blister fluid from patients with severe burns (33), supporting the involvement of this pathway in more extensive injuries. Moreover, this mechanism may become relevant even in milder burns in individuals with allergic comorbidities, potentially favoring IgE-dependent mast-cell degranulation.

Mast cells can also be activated through non-IgE pathways, including IgG immune complexes, complement anaphylatoxins (C3a, C5a), TLR ligands, cytokines such as IL-33, and neuropeptides such as Substance P (34, 35) released from peptidergic nociceptors when activated by heat. In burn injury, mast cells activation is likely driven by complement activation and DAMP-induced TLR signaling, although the precise upstream receptors remain incompletely defined. TLR activation via MyD88 adaptor proteins triggers NF-*κ*B, JNK, and p38 MAPK pathways, then sustain cytokine production and contributes to the prolonged inflammatory response characteristic of severe burns (7, 36–39).

Immediately after thermal insult (seconds to minutes), mast cells (*in vivo* and *in vitro*) discharge different inflammatory mediators such as histamine, tryptase, chymase, heparin, and preformed chemotactic factors (37, 40–44). Burn-induced histamine release may contribute to the characteristic post-burn vasodilation and vascular permeability through H1- and H4-receptor signaling, while tryptase activation of PAR-2 on nociceptors and endothelial cells contributes to edema and early nociceptor sensitization (45–49).

A second wave of mediators, emerging within 30–120 min, includes lipid-derived products such as prostaglandin E_2_ (PGE_2_), leukotrienes, and platelet-activating factor. Burn models demonstrate early increases in prostanoids and leukotrienes (7, 50, 51) which contribute to vascular leakage and nociceptor activation. While these lipid mediators arise from multiple cell types, mast cells are a significant source and are likely contributors in the early post-burn milieu (52, 53).

A third, delayed wave of newly synthesized mediators, developing from ∼2 h onward, includes TNF-α, IL-1β, IL-6, chemokines (e.g., CXCL8/IL-8), and nerve growth factor (NGF). Burn-activated mast cells upregulate cytokine and protease expression, amplifying leukocyte recruitment, promoting endothelial activation, increasing COX-2 expression, and enhancing nociceptor sensitivity. Mast cells, along with keratinocytes, monocytes/macrophages, and activated fibroblasts, also express vascular endothelial growth factor (VEGF), a potent inducer of angiogenesis (54, 55). NGF, produced by mast cells and keratinocytes and released upon thermal injury (56–59), is a key driver of persistent hyperalgesia (60, 61). These sequential mediator waves form a mast-cell–driven signaling cascade that transitions early nociceptor activation into prolonged peripheral sensitization characteristic of burn injury.

The released inflammatory mediators influence nociceptor sensitization. These mediators modulate TRPV1 channel, a molecular hub for sensitization, expressed on peptidergic nociceptors. Serotonin (5-HT) released from mast cells activates ionotropic 5-HT_3_ receptors on primary afferent terminals, causing rapid sodium influx and robust nociceptor firing (62). Histamine primarily mediates pruritus via H_1_ and H_4_ receptors on sensory neurons; in burns, its role in pain is secondary to neurogenic inflammation and vascular effects (63). Released 20-HETE acts as an endogenous TRPV1 agonist, lowering activation thresholds for heat and chemical stimuli (30) while prostaglandin E₂, via EP4 receptors, facilitates TRPV1 re-sensitization through cAMP–PKA signalling (64). Other mediators activate GPCRs, triggering PLC*β* and PKC*ε*-mediated TRPV1 phosphorylation, reducing its activation threshold to 35–37°C and converting innocuous stimuli into painful sensations; in line with this, peripheral TRPV1 blockade reduces thermal allodynia by approximately 67% in partial-thickness burn models, with TRPV1-positive fibres co-localising with infiltrating immune cells at the burn site (65). In addition to TRPV1, mast cells also modulate TRPA1 channel activity on nociceptors. In this case, tryptase activates PAR-2 receptor, promoting sustained calcium influx via TRPA1 channels, contributing to mechanical hyperalgesia (66). Consistent with this mechanism, burn injury triggers the spinal release of oxidised lipid agonists that activate both TRPV1 and TRPA1, driving post-burn mechanical and thermal allodynia (67).

Burn injury may establish a feed-forward loop between nociceptors and mast cells. Activated nociceptors release substance P (SP) and calcitonin gene-related peptide (CGRP) antidromically. SP triggers further degranulation and serotonin release through MRGPRX2/MRGPRB2 receptors on mast cells (68). Activation of this receptor mediates inflammatory mechanical and thermal hyperalgesia and is required for the recruitment of innate immune cells at the injury site, independently of the canonical NK-1R receptor (69), amplifying nociceptor excitability. CGRP enhances histamine release and vascular permeability, contributing to erythema and oedema (70); elevated plasma levels of both SP and CGRP have been documented in patients with early burn injuries, supporting a role for these neuropeptides in the neurogenic inflammatory response to thermal injury (71). During the proliferative phase of healing (days 3–21), mast cells continue to influence nociceptive signaling and pain perception through structural and signaling mechanisms. They release matrix metalloproteinases (MMPs), tissue plasminogen activator (tPA), and PAI-1, altering extracellular matrix turnover and collagen deposition, which can distort nerve terminals and promote hypertrophic scarring (72); in patients with post-burn hypertrophic scars, mast cell density is significantly elevated compared to normal skin and correlates with scar thickness and the severity of pain and pruritus (73).

Together, these rapid and sustained mast-cell responses may position them as relevant amplifiers of burn-associated pain, linking early inflammatory signaling to prolonged nociceptor sensitization.

#### 1.2.2 Natural killer cells

Natural killer (NK) cells are innate lymphocytes that recognize and eliminate infected or stressed cells without prior antigen sensitization. They express a dynamic repertoire of activating and inhibitory receptors, which integrate signals from target cells and the surrounding microenvironment to determine whether a target cell should be spared or eliminated. Upon activation, NK cells release cytotoxic granules containing perforin and granzymes, leading to programmed cell death in target cells (74).

Following thermal injury, NK cells are recruited from the circulation to damaged skin via chemokine gradients and inflammatory signals (75). However, clinical data from burn patients reveal significant alterations in NK cell number and function after major thermal trauma, including reduced cytotoxicity, altered cytokine production profiles, and impaired responsiveness to activation signals (75). These dysfunctions contribute to increased susceptibility to infections, systemic immune dysregulation, and are correlated with poor clinical outcomes in patients suffering from extensive burns (76).

Infiltrating natural killer cells become active producers of several cytokines (77) with interferon-gamma (IFN-γ) and tumour necrosis factor-alpha (TNF-α) being particularly relevant to the inflammatory and nociceptive responses in the skin (75, 78–80). Recruited NK cells are deemed to constitute an important source of IFN-γ at burn sites. This cytokine activates macrophages, enhances their pro-inflammatory phenotype, upregulates major histocompatibility complex (MHC) class II on antigen-presenting cells and facilitates antigen presentation (81). Importantly, IFN-γ can also sensitize nociceptors through the induction of downstream inflammatory mediators, thereby linking innate immune activation with nociceptive signaling as seen in neuropathic/inflammatory pain models (7).

In addition to IFN-γ, NK-derived TNF-α contributes to direct nociceptor sensitization via TNFR1 signaling, intensifying nociceptor excitability. It also amplifies inflammatory cascades, promotes endothelial activation, and facilitates leukocyte recruitment to the site of injury. In the context of severe burns, TNF-α is implicated in the development of systemic inflammatory responses, highlighting its dual role in local and systemic immune regulation (75). Beyond IFN-γ and TNF-α, activated NK cells produce GM-CSF, IL-10, and various chemokines. These mediators further contribute to the complexity of the immune landscape and may influence both inflammation and pain resolution dynamics in burn wounds.

#### 1.2.3 Dendritic cells

Dendritic cells (DCs) are central antigen-presenting cells in the skin, where they continuously monitor the local microenvironment, capturing antigens from pathogens or damaged cells to trigger the appropriate adaptive immune responses. The skin contains two principal subsets of dendritic cells: Langerhans cells (LCs) and dermal dendritic cells.

LCs are specialized DCs located in the epidermis, residing among keratinocytes. In humans, they are identified by the expression of CD1a and langerin (CD207), whereas in mice, CD207 alone serves as main marker. Notably, LCs possess the unique ability to self-renew locally under steady-state conditions, without requiring replenishment from blood-derived precursors (82–84). Depending on the severity of the thermal damage, LCs may either remain in the epidermis or migrate out of it. In cases of severe burns, the LC networks within the epidermis are often disrupted or destroyed, leading to a depletion of these cells that later regenerate either from blood-derived monocytes or from local precursors (85).

Dermal dendritic cells represent a heterogeneous population located in the dermis. In humans, the CD1c⁺ DCs are the major dermal subset and are highly efficient at stimulating T cells. Their murine counterparts are marked by CD11b expression. Another important subset includes the CD141⁺ DCs, which specialize in cross-presentation and are critical for activating CD8⁺ T cells, particularly in response to sterile injury. In mice, these cells are identified by the expression of CD8α and/or CD103 (86). Additional dermal DC subsets include CD14⁺ DCs, which are monocyte-derived and become prominent during inflammatory conditions. The equivalent population in mice are the Ly6C⁺ DCs. Finally, plasmacytoid dendritic cells (pDCs) are specialized for the production of type I interferons, playing a key role in antiviral defence. In humans, pDCs express CD123 and BDCA2 as defining molecular markers, while in mice, they are marked by B220, Siglec-H, or BST2 (87).

Burn injury induces a state of sterile inflammation, characterized by tissue damage in the absence of infection, which activates dendritic cells (DCs) through endogenous danger signals (88). These DAMPs include HMGB1, extracellular ATP, HSP70/90, uric acid, and mitochondrial DNA, which activate PRRs on DCs, leading to inflammasome and type I interferon pathway activation (89). Studies using sterile heat-injury models have demonstrated that CD8⁺ lineage dendritic cells are essential for priming CD8⁺ T-cell responses to antigens released during burn injury. Their activation requires signaling through caspase-1 and the MyD88 pathway. Experimental depletion of these CD8α⁺ DCs results in a significant impairment of the adaptive immune response to burn injury, highlighting their relevant role in post-burn immunity (90).

During the inflammatory phase of the burn injury, monocyte-derived dendritic cells, also referred to as inflammatory DCs, are rapidly recruited from the bloodstream to the site of tissue damage. These cells produce high levels of inflammatory cytokines which contribute to the local inflammatory milieu (4, 91) and modulate pain-related signaling. In this regard, DC-produced IL-1β, processed via inflammasome activation, sensitizes TRPV1 channels, enhances nociceptor excitability, and promotes prostaglandin synthesis (mainly shown in non-burn contexts) (92). TNF-α, rapidly produced at burn sites, binds TNFR1 on nociceptors, enhances sodium channel function and promotes central sensitization through glial cell activation. Sustained elevation is associated with chronic pain, but most direct links to chronic pain come from neuropathic or inflammatory models (93). In these models, IL-6, produced in response to DAMPs, activates GP130 signaling and sensitizes nociceptors via the STAT3 pathway (94). Given that burn injury has both inflammatory and neuropathic characteristics, it is conceivable that this mechanism also contributes to pain chronification in burns.

DCs also secrete chemokines with direct nociceptive effects in skin injuries. CCL2 (MCP-1), produced by DCs and other immune cells, activates CCR2 on nociceptors, directly exciting sensory neurons and contributing to inflammatory and neuropathic pain. CCL3 (MIP-1α), released by DCs and macrophages at burn sites, acts via CCR1 and CCR5 to promote nociceptor sensitization while its neutralization reduces hyperalgesia in inflammatory models (not yet studied in burns) (95). In burn injuries, CXCL1 (GRO-α), produced by keratinocytes and DCs, activates CXCR2 on nociceptors and contributes to neutrophil recruitment and peripheral sensitization (96). Conversely, nociceptors modulate DC behaviour through the release of neuropeptides and neurotransmitters. Nociceptor-derived CGRP may inhibit DC maturation and migration and reduce pro-inflammatory cytokine production (97). In contrast, substance P enhances DC maturation, cytokine production, and migration to lymph nodes, thereby amplifying adaptive immune responses. This crosstalk has been observed in inflammatory models and most likely will be present in burn injuries (98).

The spatial organization of DCs and nociceptive nerve terminals within the skin suggests a close anatomical and functional relationship. Dermal DCs are situated in proximity to peptidergic nerve fibers, while Langerhans cells extend dendritic processes among epidermal nerve endings (99, 100). This intimate arrangement facilitates bidirectional signaling between immune and sensory cells, but detailed signaling mechanisms have mainly been mapped in models of cutaneous infection or generalized inflammation (99, 101, 102). Microscopy studies provide evidence for physical interactions between DCs and nerve fibers. DC processes have been observed in direct contact with sensory terminals, and gap junctions or other intercellular communication structures may facilitate localized signalling (101). The presence of membrane-bound mediators, such as membrane TNF-α, underscores the importance of cell-cell proximity in neuroimmune communication (99).

Effects of DCs depletion have been investigated using CD11c-DTR transgenic mouse models, which allow for the conditional ablation of CD11c⁺ cells. Administration of diphtheria toxin selectively depletes these cells during the early post-burn period. DC depletion has been associated with impaired healing outcomes; specifically, delayed wound closure, with significantly slower re-epithelialization (103). Conversely, enhancement of DC populations through systemic administration of Flt3 ligand (FL) results in increased DC presence at burn sites, accelerating early wound closure, and improving tissue quality during the healing process (104). Depletion of Langerhans cells (LCs) has been shown to reduce contact hypersensitivity in other contexts; however, their specific role in burn-associated pain remains unaddressed (105).

#### 1.2.4 T-cells

T-cells, the principal lymphocytes involved in cell-mediated immunity, enhance innate defences, orchestrate adaptive responses against foreign antigens, and regulate nociception. Although αβ T cells are the most abundant T-cell population systemically, epithelial tissues, such as the skin, contain an enriched population of γδ T-cells. These γδ T-cells play important roles in regulating inflammation, maintaining epithelial homeostasis, and supporting tissue repair and regeneration (106). In healthy mouse skin, γδ T-cells constitute the dominant resident T-cell subset. These epidermal T-cells function as rapid sentinels that respond immediately to injury and help coordinate the early immune response. In contrast, human skin is populated predominantly by αβ T-cells, particularly CD8⁺ tissue-resident memory T-cells (TRM) in the epidermis, along with CD4⁺ T-cells, regulatory T-cells (Tregs), and effector-memory T-cells within the dermis (107). Although far less abundant than in mice, γδ T-cells in human skin remain functionally significant, contributing to both immune regulation and the pathogenesis of several inflammatory skin disorders (108).

Following burn injury, mouse γδ T-cells rapidly accumulate at the wound site, where they secrete chemokines that recruit neutrophils, monocytes, and later αβ T-cells. This early γδ T-cell activation shapes the initial inflammatory response and is essential for limiting neutrophil-driven tissue damage: mice lacking γδ T-cells exhibit increased mortality, exacerbated inflammation, and impaired wound repair (109, 110). Beyond the acute phase, γδ T-cells further modulate myeloid cell activity and help orchestrate the transition into the proliferative phase of healing (111, 112).

As healing progresses, αβ T cells, comprising both CD4⁺ helper and CD8⁺ cytotoxic subsets, become the predominant lymphocyte population at burn sites due to a sustained recruitment signals from injured tissue. These αβ T-cells can suppress excessive lymphocyte proliferation and produce pro-inflammatory cytokines that support early microbial control and debris clearance. At the same time, they exert regulatory functions that fine-tune the inflammatory environment, creating the conditions necessary for effective tissue regeneration (7).

The contribution of T-cell subsets to post-burn pain remains poorly defined. Most mechanistic insights derive from mouse models, whereas human data are sparse and largely descriptive. In mice, early γδ T-cell activation shapes the inflammatory milieu after injury, influencing nociceptive sensitivity. γδ T-cells release cytokines such as IFN-γ, IL-10, and particularly IL-17, which modulate neuroimmune interactions and contribute to nociceptor hypersensitivity (113). As healing progresses, naïve αβ CD4⁺ T cells primed in secondary lymphoid organs differentiate into Th1, Th2, Th17 and Treg subsets and are recruited to the burn site, where local inflammatory cues further shape their functional phenotype. Th1- and Th17-driven responses are implicated in sustaining neuroinflammation and can contribute to hyperalgesia, whereas Th2 cells and Tregs exert counter-regulatory and potentially pain-modulating effects (114). Extensive burn injury also induces prolonged αβ T-cell dysfunction, marked by reduced CD4⁺/CD8⁺ activation and dampened cytokine production, which increases infection susceptibility and may contribute to broader immune dysregulation that facilitates the transition to chronic pain.

Evidence from neuropathic and inflammatory pain models further supports a role for T-cell balance in shaping pain outcomes, with Th1/Th17 activity enhancing, and Treg activity limiting, persistent nociception. Post-burn immune responses often shift toward a Th2/Treg phenotype with elevated IL-10, which may transiently suppress nociceptive sensitization; however, persistent Th17 activity and sustained IL-17 production at some burn wounds could maintain neuroimmune signaling that promotes chronic pain (111, 115, 116). Although these mechanisms are documented in non-burn contexts, direct experimental evidence in burn injury is still limited, thus research in this direction is of great interest.

#### 1.2.5 B-cells

B-cells, traditionally recognized for their role in antibody production and systemic adaptive immunity, also participate in tissue-specific immune regulation across peripheral organs, including the skin. Skin-resident B-cells are heterogeneous, enriched in large lymphocytes and B-1 like cells that co-express IgM and CD11b are located within the dermis. There they constitute a specialized population adapted for local immune surveillance and rapid responses to tissue injury. They express elevated levels of MHC class II, CD1 molecules, and the costimulatory receptors CD80 and CD86, reflecting a high intrinsic capacity for antigen presentation to T-cells (117). Therefore, skin-resident B-cells can initiate and amplify adaptive immune responses locally within the tissue microenvironment.

Burn injury induces profound alterations in both local and systemic B-cell populations, with the nature and magnitude of these changes depending on anatomical compartment, burn severity, and time after injury. Analysis of human burn scar demonstrates progressive B-cell infiltration, with absolute numbers increasing from the third week post-injury (118). In contrast to this local expansion, burn injury produces marked disturbances in systemic immunoglobulin levels. These changes follow a characteristic biphasic pattern. In the immediate post-burn period, antigen-specific immunoglobulin production can rise briefly; in mouse models, antigen-specific IgM synthesis (e.g., against bacterial polysaccharides such as PGPS) is elevated on post-burn day 1, reflecting an early response to microbial challenge (119). However, this increase is transient. Clinical studies show that major serum immunoglobulin classes fall rapidly thereafter, with the minimum of serum gammaglobulin levels typically occurring around 48 h post-injury (120). After this early depression in immunoglobulin concentrations, each immunoglobulin class begins to recover at a different pace (120). As a result, serum Ig levels remain markedly reduced throughout the first week after injury (120, 121), and in some cases may remain depressed for several subsequent weeks (120, 122). Notably, this decline precedes substantial changes in circulating B-cell numbers, indicating that mechanisms other than simple B-cell depletion are involved. Mechanistically, this sustained depression most likely reflects a combination of impaired antibody production and ongoing antibody loss. In this context, transforming growth factor-β (TGF-β) and prostaglandin E_2_ (PGE_2_) are recognized as important regulatory mediators in the post-burn immune response and are capable of dampening B-cell function and immunoglobulin synthesis, potentially reducing the pool of antibody-secreting cells by limiting clonal expansion (123) and also suppressing some immunoglobulin synthesis (124). More consistently, data support that increased microvascular permeability after severe burns leads to enhanced extravasation, tissue sequestration, and vascular leak of plasma proteins, which promotes antibody loss and thereby further exacerbates hypogammaglobulinemia (45, 125, 126).

Recent evidence highlights a direct role for B-cells and their antibodies in pain regulation, beyond their secretion of pro- and anti-inflammatory cytokines. Although B-cell–derived cytokines can modulate nociception through pathways similar to those of other immune cells, their most distinctive contribution arises from antibody-dependent mechanisms. These include immune complex formation, engagement of Fc receptors, and activation of complement pathways. Following peripheral nerve injury, IgG antibodies accumulate within dorsal root ganglia (DRG) and spinal cord tissue, where they colocalize with sensory neurons and glial cells, an effect also observed in human DRG samples obtained from individuals with chronic pain (127). Binding of IgG to neuronal Fc*γ* receptors lowers activation thresholds and increases spontaneous firing. IgM antibodies similarly contribute by forming immune complexes that deposit in spinal tissues and activate complement. Generated C5a sensitizes neurons, macrophages, and microglia, thereby amplifying pain transmission (128). Supporting this, administration of IgG or IgM from nerve-injured animals induces allodynia in B-cell-deficient mice (127, 129, 130), whereas blockade of Fc*γ* receptors or complement signaling eliminates these effects (127). These antibody responses appear injury-specific, reflecting recognition of neoantigens generated by tissue damage. Despite the reduced Ig levels in plasma, the increased vascular permeability may facilitate antibody driven sensitization of nociceptors and spinal pain circuits.

Experimental depletion of B-cells provides strong evidence for their involvement in both the initiation and maintenance of pain in different murine models, suggesting a similar contribution in burn injury. Pharmacological studies using anti-CD20 antibodies show that B-cell removal significantly alters pain progression. A single anti-CD20 infusion at the time of peripheral nerve injury prevents the development of mechanical allodynia, whereas later administration reduces vascular and nociceptive abnormalities in complex regional pain syndrome (CRPS)-like models. Notably, anti-CD20 treatment can also gradually reverse established sensitization, indicating that B-cells contribute to the persistence of chronic pain as well as its onset (127). Genetic approaches further support a causal role for B-cells in neuropathic pain. Mice lacking mature B-cells (muMT strain) display marked protection from pain development: they do not develop allodynia after nerve injury and exhibit attenuated pain responses in fracture and disk puncture models. Passive transfer of antibodies from injured wild-type donors reinstates pain behaviors in muMT recipients, demonstrating that antibodies are sufficient to drive pronociceptive signaling. These effects are absent in FcγR-deficient mice in the context of IgG-mediated pain, underscoring the requirement for Fc receptor signaling in antibody-driven nociceptive sensitization (127, 129).

Together, these findings indicate that B-cells influence pain through mechanisms that extend beyond their classical immunological functions.

#### 1.2.6 Macrophages

Macrophages are immune cells that play a central role in host defense and tissue repair. They constitute an heterogeneous population of mononuclear phagocytes that arise from bone marrow-derived monocytes recruited to injured tissues, as well as from embryonically derived tissue-resident macrophages that persist through local self-renewal (131–133). They act as professional phagocytes, clearing pathogens and debris, and regulate inflammation through the release of cytokines and growth factors (134). In the skin, resident macrophages are crucial for barrier maintenance, inflammation initiation and wound healing (135–139).

Macrophages have traditionally been categorized into a pro-inflammatory M1 phenotype and a reparative M2 phenotype. Although this classification is useful, it represents a simplification, as macrophages *in vivo* exhibit a continuum of activation states along the M1–M2 polarization axis. Classically activated M1-like macrophages exhibit a pro-inflammatory phenotype and produce high levels of cytokines such as TNF-α, IL-1β, and IL-6, together with reactive oxygen species that contribute to pathogen killing and tissue inflammation (140–142). In contrast, alternatively activated M2-like macrophages are associated with tissue repair and resolution of inflammation, characterized by secretion of anti-inflammatory and pro-repair mediators such as IL-10 and TGF-β, which promote extracellular matrix remodeling, angiogenesis, and wound healing (137, 143, 144). Moreover, IL-4-polarized M2 macrophages can produce endogenous opioid peptides (e.g., enkephalins, endorphins, dynorphins), which have been shown to mediate sustained analgesia in models of nerve injury, linking M2-like macrophage activation to both tissue repair and pain relief (145).

Upon thermal injury, skin resident macrophages respond rapidly and persistently (146). They use several pattern-recognition receptors, to sense and respond to damage-associated molecular patterns (DAMPs) released by necrotic keratinocytes and fibroblasts (147). In the early post-burn phase, macrophages contribute to local inflammation by producing pro-inflammatory cytokines (e.g., TNF-α, IL-1β, IL-6), chemokines (e.g., IL-8), and lipid mediators such as prostaglandin E₂. These mediators promote increased vascular permeability and leukocyte recruitment in burn wound tissue, and can directly sensitize peripheral nociceptors, enhancing pain signaling (75, 148, 149).

Burn injury induces a substantial influx of inflammatory monocyte-derived (M1-like) macrophages into the wound. These pro-inflammatory macrophages expand during the first days after injury and sustain the inflammatory milieu initiated by resident macrophages, further increasing the production of reactive oxygen and nitrogen species (149). In severe burns, a delayed transition from M1-like to M2-like macrophages, together with excessive pro-inflammatory signaling, has been linked to impaired resolution of inflammation, pathological tissue remodeling, and scarring, which are in turn associated with long-term functional impairment and persistent pain (149, 150).

Macrophages play a pivotal role in nociceptor modulation through close chemical interactions with sensory neurons in the skin. They release a wide array of mediators that alter nociceptor excitability. Among the most important pro-nociceptive mediators are TNF-α, IL-1β, and IL-6. TNF-α enhances TRPV1 and voltage-gated sodium channel activity, lowering activation thresholds and promoting hyperalgesia (151, 152). Similarly, IL-1β and IL-6 activate intracellular signaling cascades such as MAPK and JAK-STAT pathways, increasing neuronal sensitivity and contributing to persistent pain (153). Chemokines such as CCL2 not only recruit monocytes to the wound but also directly sensitize neurons via CCR2 signaling (154, 155). Neurotrophic factors like NGF and BDNF further amplify nociceptive signaling by promoting nociceptor sprouting and upregulating TRPV1 expression, creating a hyperexcitable neuronal state (156, 157). Lipid mediators such as prostaglandin E2 (PGE2) also contribute by activating EP receptors on nociceptors, sensitizing TRPV1 and sodium channels and lowering pain thresholds.

Conversely, M2 macrophages exert analgesic effects by producing endogenous opioid peptides, including met-enkephalin, dynorphin A, and β-endorphin. These peptides activate peripheral opioid receptors on sensory neurons, reducing hypersensitivity. This mechanism was confirmed in neuropathic pain models where perineural transfer of M2 macrophages reversed pain in a naloxone-sensitive manner, highlighting the therapeutic potential of promoting M2 phenotypes in burn wounds (145, 158, 159).

Pain trajectories in burns closely parallel macrophage infiltration. Early dominance of M1 macrophages correlates with hyperalgesia, while later expansion of M2 macrophages may provide endogenous analgesia. Strategies that promote M2 polarization or block M1-derived mediators, such as TNF-α inhibitors or anti-NGF antibodies, could improve both healing and pain outcomes (144, 160).These findings underscore the dual role of macrophages as both drivers of pain and agents of resolution, making them a critical therapeutic target in burn injury management.

## 2 Epithelial cells alterations and nociceptive dysregulation in burn injury

**Figure 2:**
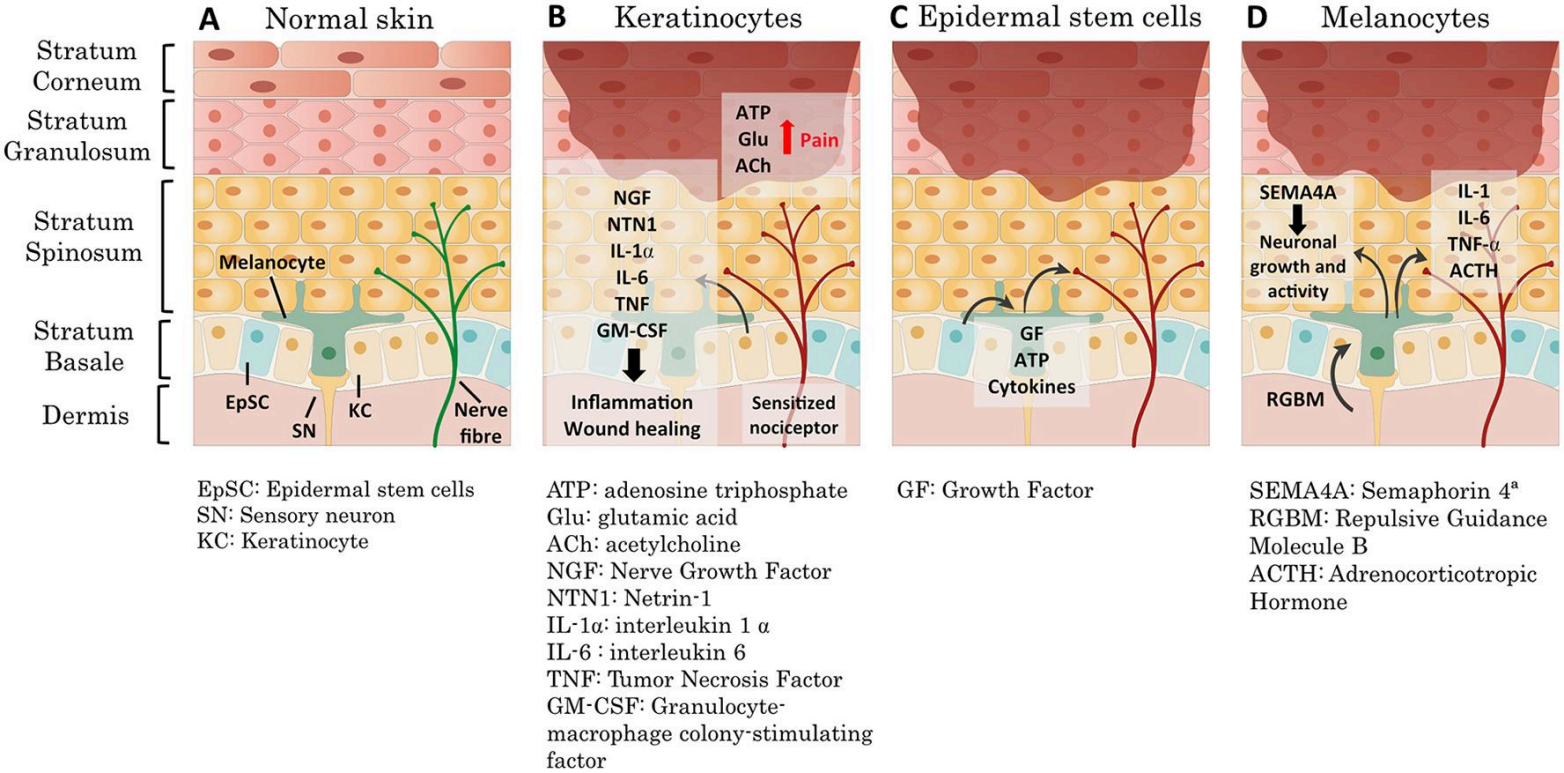
Epidermal cell–derived molecular drivers of nociceptive sensitization. The figure depicts the cellular and molecular microenvironment of the epidermis, comparing normal skin **(A)** with the skin after burn injury **(B–D)**. Following burn-induced inflammation, keratinocytes (KCs, **B**) release pro-inflammatory and neurotrophic mediators, such as ATP, IL-6, and TNF-α, which modulate nerve fiber activity. During the healing phase, epidermal stem cells (EpSCs, **C**) are activated by growth factors (GFs) to promote regeneration, while semaphorin 4A (SEMA4A) and additional signaling molecules regulate melanocyte **(D)** function and neuronal activity.

As the inflammatory phase of wound healing concludes, the process transitions into the proliferative phase, during which tissue repair is primarily driven by keratinocytes and fibroblasts that collaborate to rebuild the damaged area (161, 162). This phase is characterized by the formation of granulation tissue, cellular proliferation, angiogenesis, and deposition and remodeling of extracellular matrix components, all of which promote wound contraction and closure (163). A key event during this stage is re-epithelialization, whereby keratinocytes at the wound edge and from nearby structures migrate across the wound bed to restore the epidermal barrier, a critical step for re-establishing the skin's protective and sensory functions (164). Epithelial cells (such as regenerating keratinocytes and sensory-nerve associated structures) and mesenchymal cells (including fibroblasts and myofibroblasts) within the healing skin can contribute to increased nociceptive sensitivity, through inflammatory, neurochemical, and structural mechanisms that modulate nociceptor excitability and local nerve remodeling (165, 166) (Figure 2). Next, we describe how burn injury affects these cell types and the mechanisms through which they contribute to heightened nociception.

### 2.1 Keratinocytes

Keratinocytes are the main cell type in the epidermis (approximately 90%–95% of epidermal cells in both humans and mice), constituting the structural and functional backbone of the skin (167). They are produced from epidermal stem cells, giving rise to all layers of the epidermis. Traditionally, keratinocytes were considered passive barrier cells; however, recent studies have challenged this view, highlighting their role as sensory cells capable of modulating skin processes, including nerve fiber activity (168) contributing to nociception in some neuropathic models (169, 170).

In uninjured skin, keratinocytes sense chemical and physical damaging signals from the extracellular milieu through the expression of various ion channels, notably transient receptor potential (TRP) channels, with TRPV1, TRPA1, TRPM3, TRPV3 and TRPV4 being relevant (171–173). TRPV1 responds to noxious heat, capsaicin, and acidic pH, and is upregulated during inflammation and injury, contributing to neurogenic inflammation and pain sensitization (174). Direct evidence that keratinocyte TRPV1 activation is sufficient to induce pain *in vivo* comes from studies in which selective expression of TRPV1 in keratinocytes of TRPV1-knockout mice was enough to evoke nocifensive behavior and spinal nociceptive activation following capsaicin application (175). TRPA1, activated by cold (less than or equal to 16°C), several endogenous inflammatory mediators or irritants, enhances contact hypersensitivity and inflammatory responses (176, 177). TRPV3, responsive to innocuous warmth, promotes keratinocyte proliferation, cytokine secretion, and wound healing, via its interaction with the chloride channel ANO1 (178, 179). Alongside its role in tissue repair, TRPV3 activation triggers a massive influx of Ca^2+^ into keratinocytes, prompting the non-neuronal release of excitatory neurotransmitters such as ATP, which subsequently bind to purinergic receptors (e.g., P2X3) on adjacent free nerve endings (172), effectively amplifying nociceptive signaling and contributing to pain (180–182).

Beyond thermal and chemical sensing, keratinocytes detect mechanical stress through the force-gated ion channel PIEZO1, which is predominantly expressed at the rear of migrating keratinocytes. PIEZO1 regulates cell retraction and collective migration, affecting the speed and quality of re-epithelialization. Activation of PIEZO1 in keratinocytes can directly induce action potentials in sensory neurons (183), with relevance in neuropathic pain contexts. In wounds, PIEZO1 activity may slow wound closure, suggesting its modulation could be a therapeutic target (170).

Following a burn injury, keratinocytes undergo profound changes. Thermal damage triggers necrosis and regulated forms of cell death, including apoptosis and pyroptosis. Surviving keratinocytes engage in SNARE-mediated exocytosis to release signaling molecules such as ATP, glutamate, and acetylcholine. These mediators activate specific membrane receptors and ion channels on adjacent nociceptive fibers, promoting peripheral sensitization and amplifying nociceptive signaling (171). At the wound margins, keratinocytes become proliferative, upregulating markers such as KRT5, vimentin, and NOX2, and secreting IL-1α, IL-6, TNF-α, and GM-CSF, which contribute to inflammation, fibroblast activation, and tissue repair (184, 185). Under inflammatory and stress conditions, keratinocytes also increase production of Netrin-1, a guidance cue that modulates neurite outgrowth in regenerating sensory fibers and contributes to nociceptor hyperexcitability (186). Additionally, they influence sensory axon regeneration and pain perception through the release of nerve growth factor (NGF), which activates TrkA receptors on nociceptors via the PI3K signaling axis, promoting axon sensitization and pain behaviors (187).

Beyond their ability to regulate nerve activity through chemical mediators, keratinocytes have been shown to directly interact with intraepidermal nerve terminals. Imaging studies reveal that keratinocytes form synaptic-like contacts with sensory neurons, ensheath afferent terminals, and establish connexin43-mediated junctions, enabling bidirectional communication (188). However, transfer of small molecules through these junctions *in vivo* has yet to be confirmed and remains an active area of research (181).

In summary, keratinocytes are now recognized as thermosensory, mechanosensory and chemosensory cells that actively participate in nociception and itch signaling in addition to wound healing, with broad implications for both cutaneous sensation and regenerative medicine (171, 183, 188, 189). It should be noted, however, that most mechanistic evidence derives from non-burn settings, and whether burn-injured keratinocytes engage these same pathways at comparable magnitudes remains to be established.

### 2.2 Epidermal stem cells

Epidermal stem cells are a specialized population of cells derived from the ectoderm that primarily reside in the basal layer of the epidermis and in hair follicles, where they represent 1%–7% or 2%–5% in mouse and human basal layer keratinocytes, respectively. They can self-renew and differentiate into various types of epidermal cells, playing a crucial role in maintaining skin homeostasis (190).

Burn injuries cause extensive damage to the epidermis, often disrupting the native stem cell niches essential for skin regeneration. Classical epidermal stem cells, marked by KRT14⁺ and TP63⁺ expression, reside in the basal layer of the interfollicular epidermis and are highly sensitive to thermal damage (191, 192). In partial-thickness burns, surviving cells proliferate and differentiate to restore the epidermal barrier, aided by inflammatory cytokines such as IL-1 and TNF-α (193). In deeper burns, their loss impairs re-epithelialization, necessitating therapeutic interventions like skin grafts (194). Lgr6⁺ epidermal stem cells, located in the isthmus of hair follicles and interfollicular regions, are primed for injury response and exhibit nerve-dependent activation, contributing to epidermal and appendage regeneration (195). In contrast, Lgr5⁺ stem cells, found in the bulge region of hair follicles, are more quiescent and primarily support hair follicle cycling; their contribution to burn wound healing is limited unless their niche remains intact (196).

Recent research has revealed a dynamic interaction between epidermal stem cells and sensory nerve fibers that significantly influences skin regeneration. Sensory nerves release neuropeptides and growth factors, such as substance P and CGRP, that modulate the local microenvironment, promoting stem cell activation, proliferation, and migration during wound repair (197). Notably, Lgr6⁺ epidermal stem cells have been shown to rely on intact sensory innervation for effective re-epithelialization, suggesting the existence of a perineural niche that supports stem cell function (195).

Emerging evidence suggests that epidermal stem cells may play an active role in modulating nociception through direct and indirect interactions with sensory neurons. Beyond their regenerative functions, these cells can influence nociceptive signaling by releasing neuroactive molecules, including ATP, growth factors, and cytokines, that sensitize nearby nerve endings (198). Experimental models have demonstrated that stimulation of epidermal cells can enhance calcium influx in adjacent sensory neurons, leading to increased nociceptor sensitivity and behavioural responses. Additionally, the structural ensheathment of sensory terminals by epidermal cells, with physical contact points including gap junction proteins like Connexin43 (188), may facilitate neuronal activation, suggesting a functional neuro-epithelial interface. However, evidence for similar direct effects in humans remains much more limited. Most human data stem from organoid co-culture and diagnostic tissue imaging, with systematic and functional demonstration of direct stem cell-nerve crosstalk still emerging.

### 2.3 Melanocytes

Melanocytes are neural crest derived cells located in the basal layer of the epidermis, primarily responsible for melanin synthesis and photoprotection (199). Beyond pigmentation, melanocytes exhibit neuroendocrine and immunomodulatory functions that may influence skin homeostasis, wound healing, and potentially nociception and pain (200, 201).

Melanocytes display dendritic morphology with long, branched projections that intermingle with keratinocytes, facilitating the transfer of melanosomes (202). They are located in close proximity to intraepidermal free nerve endings (FNEs), particularly C fibers and Aδ fibers. While evidence suggests functional interactions via paracrine signaling between melanocytes and sensory neurons, the presence of synaptic-like structures *in vivo* remains under investigation (203). In this paracrine crosstalk, sensory neurons secrete Repulsive Guidance Molecule B (RGMB), which appears to support melanocyte survival, dendrite extension, and melanin production (204). Conversely, melanocytes release Semaphorin-4A (SEMA4A), which can modulate neuronal growth and activity, particularly under UV-B stimulation (205).

In burn injury, melanocyte activity and melanogenesis are profoundly affected, frequently leading to changes in skin pigmentation (206–208). Post-inflammatory hyperpigmentation (PIH), a common sequela of burn wound healing, arises from increased melanin production and export triggered by local or systemic inflammatory responses (207). Both hyper- and hypopigmented regions often contain similar numbers of melanocytes, but these cells can exhibit differing levels of activation and dendritic activity (209, 210).

Melanocytes also secrete mediators such as IL-1, IL-6, TNF-α, α-MSH, and ACTH, which can act on melanocortin receptors (MC-Rs) expressed in melanocytes and sensory neurons. These interactions may influence pigmentation, inflammation, and nociceptive signaling (211). MC1R, highly expressed in skin melanocytes, monocytes, and macrophages, has been implicated in acute thermal and inflammatory pain. MC1R activation can reduce inflammatory pain responses, whereas loss-of-function polymorphisms may increase pain sensitivity (212). MC3R and MC4R are expressed in peripheral neurons, and animal studies suggest their activation by melanocortin agonists (α-MSH, ACTH) can induce hyperalgesia and antagonize opioid analgesia. Consequently, MC4R antagonists reduce inflammatory pain in experimental models, though the extent to which this applies to humans remains under investigation (213).

Under inflammatory conditions, melanocytes also express pattern recognition receptors (PRRs) (214) and MHC-II (215), allowing them to sense danger signals and release cytokines such as IL-6, and TNF-α which may contribute to nociceptor sensitization and neuroimmune interactions. The close interactions between melanocytes, sensory neurons, and immune cells position melanocytes as potential modulators of neuroinflammation and peripheral sensitization, although the full extent of these effects in humans is still being elucidated (216).

Taken together, the potential involvement of melanocytes in burn-associated pain is an intriguing possibility, but one that currently rests on indirect evidence. Most of the studies linking melanocytes to sensory neuron function were conducted under homeostatic or UV-irradiated conditions. Direct evidence that post-burn changes in melanocyte activity meaningfully influence nociception is lacking, and this remains one of the least explored aspects of burn sensory pathophysiology.

### 2.4 Fibroblasts

Fibroblasts are mesenchymal cells derived from the embryonic mesoderm, and they are the dominant resident cell type in the dermis, comprising approximately 70%–80% of dermal stromal cells (217). They are found primarily in the papillary and reticular layers of the dermis, which lie beneath the epidermis (218).In healthy skin, fibroblasts primarily maintain the extracellular matrix (ECM) and contribute to tissue homeostasis (218). During the wound healing cascade, a critical biochemical shift occurs in the extracellular matrix composition. In the proliferative phase, fibroblasts predominantly synthesize type III collagen to establish a provisional scaffold. Conversely, the remodeling phase is characterized by a gradual replacement of these fibers with type I collagen, which provides superior mechanical strength and restores the tissue's structural integrity (219).

Although they do not actively regulate nerve activity, they support the survival and structural integrity of sensory nerve fibers by secreting neurotrophic factors such as nerve growth factor (NGF) and brain-derived neurotrophic factor (BDNF) (220, 221). These molecules help maintain the function of nerve fibers (220).

During wound healing, including in burn injuries, fibroblasts become highly activated in response to both biochemical signals and mechanical (218). Key activating signals include TGF-β, platelet-derived growth factor (PDGF), and IL-6, which are secreted by platelets, macrophages, keratinocytes, and other immune and endothelial cells in the wound microenvironment (222). These molecules initiate fibroblast recruitment, proliferation, and differentiation into myofibroblasts, which are characterized by α-SMA expression and enhanced contractile activity (218, 223).

Fibroblasts also respond to mechanical cues such as tissue tension, stiffness, and shear stress, which they detect through focal adhesions involving integrins (224, 225). These structures link the ECM to the cytoskeleton and activate mechanotransduction pathways, including RhoA/ROCK, YAP/TAZ, and focal adhesion kinase (FAK), driving cytoskeletal remodeling and gene expression changes (225, 226). This mechanosensing capacity enables fibroblasts to adapt to the physical properties of the wound environment and contributes to their transition into myofibroblasts (218).

Recent preclinical models and transcriptomic studies have suggested that activated fibroblasts can directly interact with nerve fibers during injury. They may acquire neural-like properties, express genes involved in axon guidance, and physically influence nerve regrowth (218). In inflammatory conditions, specific fibroblast subsets promote increased local innervation and neuro-immune crosstalk, contributing to increase pain sensation. More specifically, in animal models, activation of fibroblasts expressing TLR4 promotes mechanical sensitization and hyperalgesic priming, favouring the transition from acute to chronic pain (227). This fibroblast–nerve fiber interaction during wound healing is emerging as a relevant contributor to peripheral sensitization, which serves to protect wound closure but, in some patients, contributes to chronic pain and neuropathic features often observed in burn survivors.

### 2.5 Merkel cells

Merkel cells are specialized epithelial cells predominantly located in the basal layer of the epidermis. They originate from the epidermal lineage during development and are maintained in adult skin through continuous regeneration from epidermal stem cells (228). While distributed throughout the skin, they exhibit regional variations in density, with higher concentrations in areas requiring fine tactile discrimination. Beyond cutaneous locations, Merkel cells are also found in the oral mucosa, where their loss has been associated with pathological conditions such as oral lichen planus and hyperkeratosis (229).

Electrophysiological studies have demonstrated that Merkel cells are excitable cells capable of generating Ca^2+^-dependent action potentials (230) and responding to mechanical stimuli (231). They are essential mechanotransducers for touch sensation, with the force-gated ion channel Piezo2 playing a critical role in this function (231). Merkel cells interact closely with sensory nerve terminals to form the Merkel cell–neurite complex (also known as the Merkel disc), which consists of a Merkel cell in close apposition to the expanded terminal of a slowly adapting type I (SAI) Aβ afferent fiber (232).

The spatial organization of these complexes varies by region: in glabrous skin, they are organized into touch domes and epidermal ridges, whereas in hairy skin, they are associated with hair follicles and other specialized touch-sensitive structures (233–235). The interface between the Merkel cell and the nerve terminal exhibits features of a chemical synapse, including synaptic vesicles within the Merkel cell and postsynaptic densities in the nerve terminal (236).

Molecular studies indicate that Merkel cells express several excitatory neurotransmitters released upon mechanical stimulation to mediate the rapid depolarization of nerve endings (235, 237). Primary candidates for this communication include 5-hydroxytryptamine (5-HT), norepinephrine (NE), and glutamate (Glu). Notably, research is divided on the primary transmitter: some studies indicate that only 5-HT evokes action potentials in SAI mechanoreceptors (237), while others using *ex vivo* mouse skin-nerve preparations demonstrate that NE, but not 5-HT or dopamine, evokes these potentials (238). Consequently, the precise nature of chemical communication within the complex, whether primarily serotonergic or adrenergic, remains to be fully clarified.

As with other skin cell types, burn injury leads to the destruction and significant loss of Merkel cells in the affected region (239). However, the role of Merkel cells in pain is less understood than that of other cutaneous cell types. Given their high content of neurotransmitters and algogenic substances, it is highly plausible that the destruction of Merkel cells contributes to acute pain shortly after injury; however, this hypothesis requires further empirical validation.

Because the ablation of Merkel cells reduces SAI mechanotransducer activity (230), burn-induced destruction of these complexes likely contributes to the regional loss of touch sensation observed in full-thickness burn injuries. Contrary to other epidermal cell types, Merkel cells are notably infrequent or absent in healed skin grafts and burn scars (239, 240). This reduction in touch-sensitive endings may contribute to mechanical allodynia (230) through the impaired “gating” of nociceptive signals by SAI mechanoreceptors at the spinal cord level. However, the SAI contribution may be limited, as mechanical allodynia is predominantly mediated by rapidly adapting mechanoreceptors (RAMs) and D-hair receptors in mice (241).

Finally, while the direct regulation of nociceptors by Merkel cells has not yet been demonstrated, their release of signaling molecules, including histamine, ATP, and noradrenaline, suggests they likely influence the functional properties of nearby sensory afferents beyond traditional mechanoreceptors.

## 3 Conclusion and perspectives

Burn injuries represent one of the most complex forms of cutaneous trauma. The depth, extent, and nature of the thermal insult profoundly shape patient outcomes, ranging from mild, transient discomfort to life-threatening systemic complications, permanent functional impairment, and death. Pain is one of the prevalent symptoms accompanying burn injury. It spans a spectrum, from mechanical hyperalgesia to extreme allodynia necessitating sedation to tolerate routine wound care. Importantly, a subset of burn survivors develops chronic pain with neuropathic features, underscoring the biological complexity of burn injury and the incomplete recovery of normal nociceptive processing (242).

A critical but often underappreciated dimension of burn pathophysiology is the biological heterogeneity of the injuries themselves. Thermal, chemical, and electrical burns differ substantially in their mechanisms of tissue damage, depth of penetration, and systemic consequences, and are therefore likely to engage cutaneous cell populations and nociceptive pathways in fundamentally distinct ways. Thermal burns primarily cause cellular coagulation necrosis, the extent of which depends directly on the temperature and duration of contact (243). Chemical burns differ substantially: while acids similarly produce coagulation necrosis, alkalis cause liquefaction necrosis by dissolving proteins and lipids, enabling much deeper and ongoing tissue penetration (244). Electrical burns present a distinct challenge, as energy is converted into heat via the Joule effect, producing damage that is frequently deep and systemic, disproportionately affecting low-resistance tissues such as nerves and blood vessels, often beneath apparently intact skin (245). The mechanistic evidence reviewed here derives predominantly from thermal burn models, and direct extrapolation to chemical or electrical injuries should therefore be made with caution. Even within thermal burns, injury depth is a key determinant of which cell populations are preserved or destroyed: superficial injuries may spare the dermis and its resident immune and stromal cells, whereas full-thickness burns eliminate the entire skin architecture simultaneously, abolishing keratinocyte signaling, stem cell niches, Merkel cell complexes, and dermal fibroblast networks in a single insult. The extent of body surface area involved adds yet another layer of complexity, as large burns trigger systemic immune dysregulation that reshapes the local wound microenvironment in ways that focal injury models cannot fully capture. Across all these etiologies, burn depth ultimately determines which local cellular populations are destroyed or preserved, directly shaping the pattern of immune activation and nociceptive dysregulation that follows (243). This inherent complexity underscores how much remains to be understood, and highlights the need for studies that go beyond wound healing outcomes to explicitly address nociception and pain as primary endpoints.

Although burn wounds share common inflammatory features with other injuries, burn-associated inflammation is qualitatively and quantitatively distinct. The unique zonal architecture of burn wounds, the massive release of damage-associated molecular patterns, vascular compromise, and the frequent presence of systemic inflammatory responses together generate a neuroimmune environment that is not readily comparable to other wound types (4, 246). Despite these differences, mechanistic understanding of burn-associated pain has lagged behind that of wound healing, and many current concepts still rely on extrapolation from simple inflammatory or neuropathic pain models rather than direct evidence from burn injuries.

The focus of this review has been on the diverse non-neuronal cell types that modulate sensory neuron function, particularly nociceptors, during burn injury and healing with emphasis on the acute burn response, as burn-specific mechanistic data for later healing stages remain scarce. Immune cells and skin-resident cells, including mast cells, macrophages, lymphocytes, keratinocytes, fibroblasts, melanocytes, and epidermal stem cells, have the capacity to shape nociceptive signaling through cytokines, chemokines, neurotrophins, lipid mediators, neuropeptides, and ion-channel modulation (247, 248). However, a striking conclusion emerging from this synthesis is the scarcity of burn-specific mechanistic data. This gap is relevant given that experimental manipulation of immune and epidermal cell populations can increase or reduce nociception in animal models. Establishing a detailed and time-resolved understanding of how individual cell types contribute to burn-induced nociception could pave the way for mechanism-based strategies to manage subacute pain during wound cleaning and healing. Improved pain control at these stages may, in turn, reduce the risk of pain chronification, an outcome that remains poorly predicted and insufficiently prevented.

Beyond acute inflammation, the long-term sensory landscape following burn injury is likely shaped by aberrant reinnervation and epigenetic priming. Unlike other forms of cutaneous trauma, the extensive destruction of dermal layers triggers disordered axonal sprouting, in which nascent nerve endings, hypersensitized by a persistent pro-nociceptive microenvironment, may perpetuate pathological excitability. This process may be further amplified by a form of “local memory” within skin-resident cells, such as keratinocytes and fibroblasts, which may undergo epigenetic modifications that sustain heightened sensitivity long after wound closure (249, 250). Understanding these complex epigenetic alterations in damaged tissue may help to explain why some patients transition to chronic, neuropathic post-burn pain, whereas others achieve sensory resolution.

A notable limitation in the field is the insufficient focus on pain assessment in burn models. While numerous animal models of burn injury exist, most were developed to study wound healing and systematically neglect pain as a primary endpoint. Injury induction methods vary widely, lack standardization, and often conflict with increasingly stringent ethical requirements for animal research (251, 252). There is therefore a need to develop refined, pain-centric burn models that minimize animal suffering while faithfully reproducing the key biological and sensory hallmarks of burn injury. Such models would enable systematic investigation of pain across healing phases, including the poorly studied pain associated with scar remodelling and tissue reinnervation, and would allow mechanistic dissection of pathways leading to chronic pain.

Sex-dependent differences represent another largely underexplored dimension of burn pain. Given the strong inflammatory component of burn injury and the well-established modulation of immune responses by sex hormones, it is plausible that pain trajectories, recovery dynamics, and immune-neural interactions differ between males and females (253–256). In addition, sex-specific alterations in inflammatory resolution programs and in skin cellular populations may further contribute to divergent pain outcomes, but these aspects have so far been only marginally examined in both clinical and preclinical studies.

Pain chronification is not an inevitable outcome of burn injury: while many patients experience progressive pain resolution, a substantial subset develop persistent pain with neuropathic characteristics that endure well beyond wound closure (13, 257). The factors driving this divergence remain poorly defined, with candidate contributors including injury severity and distribution, peripheral neuroimmune signaling, central sensitization, pre-existing comorbidities, and psychosocial context. Retrospective clinical data suggest that larger total body surface area, deeper burns, and more complex reconstructive courses are associated with a higher risk of chronic pain, but prospective, mechanistically informed human studies remain scarce (258). Recent syntheses consistently highlight the paucity of longitudinal work that couples detailed pain phenotyping with biological, neurophysiological, and imaging markers in burn cohorts (5).

Addressing these gaps will require carefully designed human studies that integrate standardized burn descriptors, quantitative sensory testing, scar and functional metrics, psychological assessments, and peripheral and central biomarkers to delineate trajectories toward recovery vs. chronification. In parallel, animal models, better suited to invasive circuit-level interrogation, should be leveraged to define burn-specific alterations in spinal and supraspinal pain pathways, with particular attention to microglial and astrocytic activation, maladaptive synaptic plasticity, and changes in descending modulation that favor persistent pain (28, 259). Coordinated longitudinal human research and mechanistically targeted preclinical work are essential to move from descriptive epidemiology toward a causal, neuroimmune framework that can support rational, mechanism-based interventions to prevent or reverse burn-related pain chronification.

Taken together, these considerations underscore the urgent need to reorient burn research toward pain as a key pathological process rather than a secondary consequence of tissue injury. Advancing our understanding will depend on deeper investigation of peripheral neuroimmune interactions, the standardization of pain-relevant burn models, the systematic inclusion of sex as a biological variable, and the integration of peripheral and central mechanisms. These advances have the potential to transform pain management strategies, reduce suffering, and mitigate the long-term emotional and functional burden borne by burn survivors.
